# Metabolomic Analysis of Fission Yeast at the Onset of Nitrogen Starvation

**DOI:** 10.3390/metabo3041118

**Published:** 2013-12-13

**Authors:** Kenichi Sajiki, Tomáš Pluskal, Mizuki Shimanuki, Mitsuhiro Yanagida

**Affiliations:** Cell Unit, Okinawa Institute of Science and Technology Graduate University (OIST), 1919-1 Tancha, Onna-son, Okinawa 904-0495, Japan; E-Mails: pluskal@oist.jp (T.P.); mizuki.shimanuki@oist.jp (M.S.)

**Keywords:** fission yeast, nitrogen starvation, metabolomics, 2-oxoglutarate, trehalose, AICAR, ergothioneine

## Abstract

Microorganisms naturally respond to changes in nutritional conditions by adjusting their morphology and physiology. The cellular response of the fission yeast *S. pombe* to nitrogen starvation has been extensively studied. Here, we report time course metabolomic analysis during one hour immediately after nitrogen starvation, prior to any visible changes in cell morphology except for a tiny increase of cell length per division cycle. We semi-quantitatively measured 75 distinct metabolites, 60% of which changed their level over 2-fold. The most significant changes occurred during the first 15 min, when trehalose, 2-oxoglutarate, and succinate increased, while purine biosynthesis intermediates rapidly diminished. At 30–60 min, free amino acids decreased, although several modified amino acids—including hercynylcysteine sulfoxide, a precursor to ergothioneine—accumulated. Most high-energy metabolites such as ATP, S-adenosyl-methionine or NAD^+^ remained stable during the whole time course. Very rapid metabolic changes such as the shut-off of purine biosynthesis and the rise of 2-oxoglutarate and succinate can be explained by the depletion of NH_4_Cl. The changes in the levels of key metabolites, particularly 2-oxoglutarate, might represent an important mechanistic step to trigger subsequent cellular regulations.

## 1. Introduction

Microorganisms naturally respond to changes in nutritional conditions by adjusting their morphology and physiology. The fission yeast *Schizosaccharomyces pombe*, a popular model organism in cell cycle research, exhibits a remarkable phenotype under nitrogen starvation (hereafter N-starvation). Following the withdrawal of the nitrogen source (NH_4_Cl) from the culture medium, *S. pombe* cells exit the cell cycle, inhibit cellular growth and enter the G0 phase, thus providing an excellent model to study cellular quiescence [1].

The adaptation to N-starvation occurs in several stages. In the initial stage (within the first ~8 h), the cell size is reduced by two subsequent cell divisions, consequently forming small and round cells [2]. Following the two rounds of division, the cell cycle is arrested pre-replicatively in an ‘uncommitted’ G1 phase [3]. During this period, cells may undergo meiosis and produce spores, providing that a partner of the opposite mating type is available. In the absence of a mating partner, the cells lose the ability to mate after 12 h and commit to the G0 phase [4]. Within 24 h of N-starvation, the cellular volume, mRNA and protein content are reduced to about 55, 20, and 50% of the vegetative cell content, respectively [5]. Fully adapted G0 cells can survive for months in the N-starvation culture medium, exhibiting increased stress resistance [6,7].

To elucidate the molecular mechanisms of the N-starvation effect in *S. pombe*, a number of genetic, transcriptomic and proteomic studies were conducted [2,5,8,9,10,11]. We previously reported 33 “super-housekeeping” genes required for N-starvation-induced quiescence [10]. Microarray analysis showed alterations in the transcriptional profiles of almost all genes immediately after N-starvation [8]. About 900 transcripts (~19% of ~4,600 detected), half of which corresponded to stress-responsive genes, were up- or down-regulated more than 3-fold. Such immense change in gene expression occurred as early as one hour after N-starvation, indicating the molecular events occurring during the first hour might be critical for the cellular adaptation. Kristell *et al*. reported >2-fold alteration of 229 transcripts within 20 min after N-starvation, accompanied by significant changes of chromatin structure in certain genomic regions [9]. Transcription of stress-related genes increased transiently up to 2 h after N-starvation, followed by a decrease afterwards. On the contrary, transcription of growth-related genes decreased immediately after N-starvation [5]. Certain transcriptional responses, however, occurred only at a later time. For example, the induction of meiosis-related genes regulated by the transcription factor Ste11 took place about 3 h after N-starvation [8]. On the proteome level, 47% of all detected proteins changed their copy numbers over 2-fold and an extensive shift from growth-related proteins to stress-responsive proteins was observed 24 h after N-starvation [5].

N-starvation is ultimately a metabolic condition. Therefore, complete understanding of its effect cannot be achieved without studying the intracellular metabolome. In addition, the most immediate response to N-starvation might not be mediated on the transcriptional level, as the transcription/translation machinery responds with an inevitable delay. In this study we carried out metabolomic analysis of *S. pombe* cells during short time course immediately following N-starvation. We focus on the time period of 1 h, before any obvious morphological changes in the cells can be observed. It should be emphasized that the two cell divisions, which represent the key steps in cellular adaptation to N-starvation in *S. pombe*, occur only after this time period. We thus aspire to characterize the direct and immediate metabolic response to N-starvation.

## 2. Results and Discussion

### 2.1. Increase in Cell Size and Number Shortly after N-Starvation

**Figure 1:**
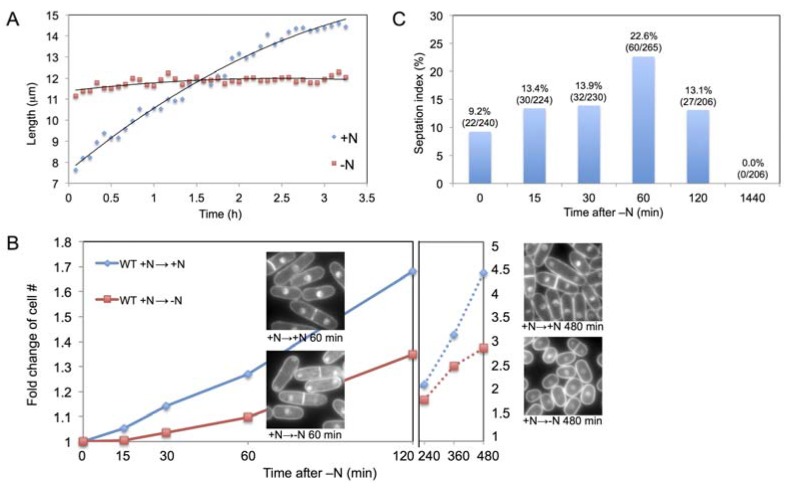
(**A**) Cell length measurement in the +N or –N medium. +N cells were incubated in EMM2; –N cells were transferred from EMM2 to EMM2-N; (**B**) Cell number measurement after medium change. Cells incubated initially in EMM2 were transferred to EMM2-N or fresh EMM2 medium at 0 min. The cell concentration was measured and plotted as a fold change against 0 min. DAPI images of cells at 60 min and 480 min are shown; (**C**) Septation index of *S. pombe* cells in asynchronous cell culture after the medium change from EMM2 to EMM2-N.

Immediately after the shift to N-starvation, *S. pombe* cells rapidly inhibit their growth (cell length increase), as observed from single cell length measurements (Figure 1A). The length of a N-starved cell increased only about ~1 µm per division cycle, considerably less than that of a vegetative cell, about 7 µm. Such result is consistent with the previously reported swift reduction in growth-related transcripts as well as proteins [5]. Cell division was transiently arrested for 15 min after N-starvation (Figure 1B). After 15 min, the cell number started to increase gradually, although considerably slower than that of vegetative cells. Interestingly, the septation index increased 15 min after N-starvation, indicating delayed cytokinesis (Figure 1C). 60 min after N-starvation, septation index reached above 20%, while cell number increased only 10%. After 120 min the cell number increase accelerated and septation index returned to ~13%. Eventually, after the full adaptation to N-starvation (24 h), septation index became 0%.

### 2.2. Time Course Metabolomic Analysis and Data Reproducibility

**Figure 2:**
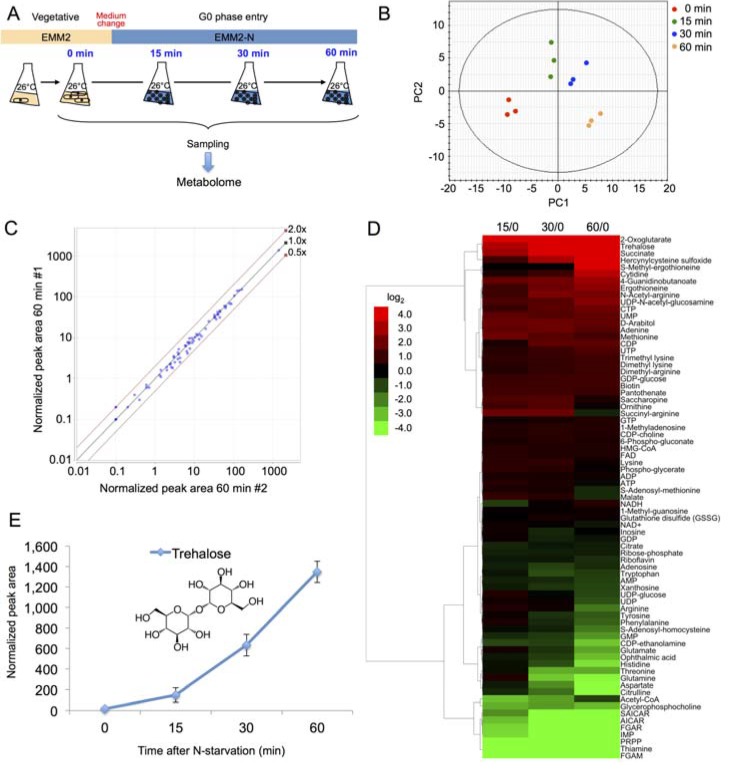
(**A**) Scheme of the time course metabolome experiment; (**B**) Principal component analysis (PCA) was conducted for the entire dataset of 75 identified metabolites (Supplementary Table S1) using the SIMCA-P+ software (Umetrics Inc., Umeå, Sweden). A scatter plot of the scores in the first two principal components is shown, with samples color-labeled according to time points; (**C**) Two normalized peak areas of all metabolites from two different samples taken at the same time point were plotted to demonstrate the data reproducibility; (**D**) The time course change of the peak areas of 75 metabolites in log_2_ values was clustered hierarchically by a complete linkage method using the Cluster 3.0 software ver. 1.50 [12] and visualized as a tree view image by the Java TreeView software ver. 1.1.6r2 [13]. (**E**) The time course change of the peak area of trehalose in cells after N-starvation.

To obtain metabolomic data sets, three independent wild-type *S. pombe* cultures were cultivated at 26 °C in liquid EMM2 medium to mid-log phase (5 × 10^6^ cells/mL). Cells were collected by vacuum filtration and shifted to EMM2-N medium (EMM2 lacking NH_4_Cl) to induce N-starvation. Aliquots of 40 mL were taken from each culture at the time points of 0 (corresponding to the vegetative culture before medium shift), 15, 30 and 60 min. Each aliquot was immediately quenched and used to prepare metabolite extracts. The experiment design is schematized in Figure 2A. Two internal standards, PIPES and HEPES, were spiked into each sample prior to metabolite extraction.

The metabolite extracts were separated by liquid chromatography on the ZIC-pHILIC column (Merck SeQuant, Umeå, Sweden) and measured on the LTQ Orbitrap mass spectrometer (Thermo Fisher Scientific, Waltham, MA, USA) in both positive and negative ionization modes. Raw mass spectra were analyzed by the MZmine 2 software [14]. Over ten thousand distinct peaks were detected in all samples. Principal component analysis calculated from the peak areas of all detected metabolites could clearly differentiate the triplicates of samples obtained at the same time point, as shown in the 2D plot of the first two principal components (Figure 2B).

Among the detected peaks, 75 individual metabolite signals were identified, mainly by comparing their *m/z* values and retention times with authentic pure standards. For 4 compounds, however, pure standards were not available; those were thus identified either by their MS/MS spectra (FGAR, SAICAR, and hercynylcysteine sulfoxide; MS/MS spectra shown in Supplementary Figure S1), or solely by the m/z value in the case of FGAM. The peak areas of the identified metabolites were measured and normalized by the peak areas of the spiked internal standards. The reproducibility of metabolite peak areas was very good between the samples prepared independently at the same time point, as shown in Figure 2C.

### 2.3. Partial Remodeling of the Cellular Metabolome after N-Starvation

The time course results of all detected metabolites are summarized in Figure 2D (complete numerical results in upplementary Table S1). During the 60 min, 6 metabolites (2-oxoglutarate, cytidine, hercynylcysteine sulfoxide, S-methyl-ergothioneine, succinate and trehalose) increased over 10-fold and 11 metabolites (AICAR, aspartate, citruline, FGAM, FGAR, glutamine, histidine, IMP, PRPP, SAICAR and thiamine) decreased over 10-fold. Interestingly, the level of high-energy compounds such as adenosine triphosphate (ATP), S-adenosyl methionine (SAM) or nicotinamide adenine dinucleotide (NAD^+^) remained relatively stable during the time course, suggesting the cellular energy pool was not impaired during the initial stage of N-starvation (Figure 2D, center area). Overall, peak areas of 29 of the 75 identified metabolites (38.7%) remained within two-fold change in peak area.

A single metabolite showing the most striking accumulation during the time course was trehalose (~80-fold increase after 60 min; Figure 2E). A similar result was previously reported in *Saccharomyces cerevisiae* [15]. Trehalose is a membrane and protein stabilizer produced by many microorganisms under stress [16]. In *S. pombe*, trehalose biosynthesis pathway starts with the trehalose-phosphate synthase enzyme Tps1. The *tps1* gene belongs to the group of core environmental stress response (CESR) genes, activated under non-specific stress conditions [17]. We have previously reported trehalose accumulation under heat shock [18] and glucose starvation [19]. Apart from the immediate protective and stabilizing function, trehalose might play an important role as a nutrient stock during months-long cellular quiescence, as it can be broken down into two molecules of glucose by the trehalase Ntp1.

### 2.4. Rapid Metabolic Response within 15 min

**Figure 3:**
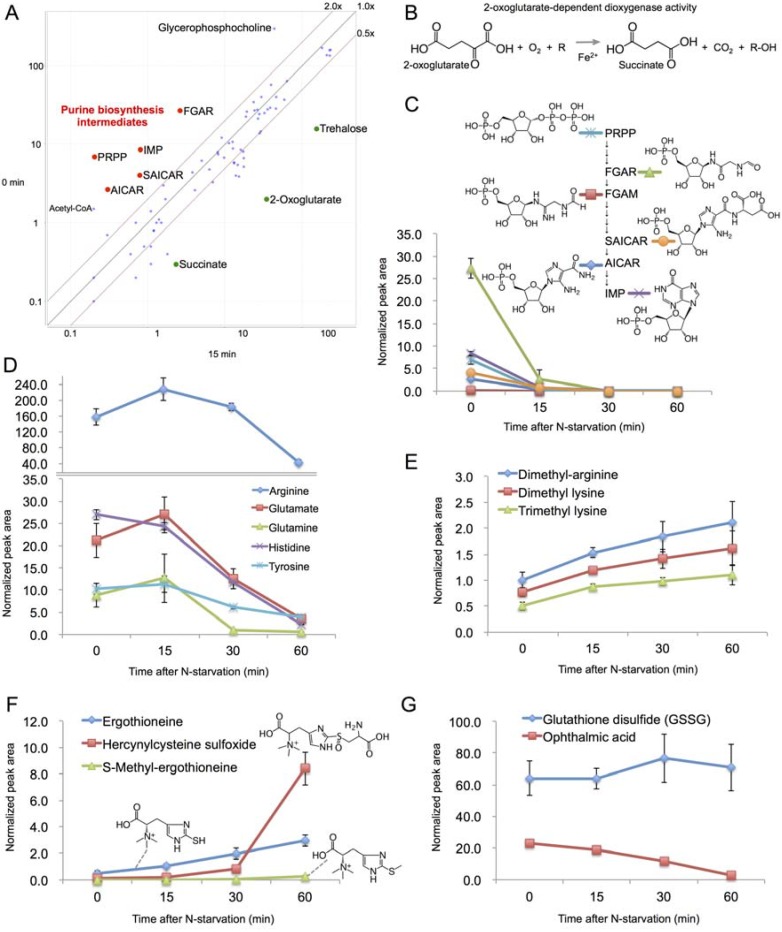
(**A**) A scatter plot comparing the normalized peak areas of all metabolites at 0 and 15 min after N-starvation (values represent averages of 3 samples); (**B**) A schema of 2OG-dependent dioxygenase reaction; (**C**–**G**) The time course change of the normalized peak areas of purine biosynthesis intermediates (**C**), amino acids (**D**), modified amino acids (**E**), ergothioneine and related metabolites (**F**) and glutathione and ophthalmic acid (**G**) in cells after N-starvation.

The changes of metabolites in 15 min after N-starvation are plotted in Figure 3A. During this interval, 2-oxoglutarate (2OG) and succinate sharply increased. Both are intermediates in the TCA cycle, however the amount of citrate remained rather constant, suggesting this effect might not be related to TCA cycle activity. 2OG is required for ammonia assimilation in *S. pombe* by glutamate synthase [20], depletion of ammonia might thus lead to the accumulation of 2OG. The increase in succinate might be a direct consequence of 2OG accumulation, as 2OG can be converted to succinate by 2-oxoglutarate oxygenases [21] (Figure 3B). This reaction requires O_2_ as a cofactor, suggesting that oxygen requirement at the onset of nitrogen starvation might change.

On the other side, PRPP (5-phospho-ribose 1-diphosphate), FGAR (N-formylglycinamide ribonucleotide), FGAM (N-formylglycinamidine ribonucleotide), SAICAR (N-succinocarboxamide-5-aminoimidazole ribonucleotide), AICAR (aminoimidazole-4-carboxamide ribonucleotide) and IMP (inosine-phosphate) decreased dramatically in 15 min and later became completely undetectable (Figure 3C). These metabolites are intermediates in the purine biosynthesis pathway [22]. In fission yeast, this pathway is mediated by *ade* genes and the transcripts of *ade1*, *3*, *4*, *5*, *6*, *7*, *8*, *10* genes were all down-regulated after N-starvation [5]. Following the removal of ammonia from the culture medium, some inhibition of the biosynthesis of nitrogen-rich compounds like purines might be expected. Nevertheless, the speed and scale of the depletion of these compounds is noteworthy, considering there was no visible change in cell morphology at this time point. Contrary to the purine intermediates, glycerophosphocholine decreased ~7-fold in the first 15 min (Figure 3A), but its level remained stable afterwards. Glycerophosphocholine is a product of phospholipid catabolism by phospholipases and an important osmolyte [23]. The change in its level might thus reflect the decrease in osmotic pressure due to the missing NH_4_Cl component in the culture medium.

### 2.5. Changes in Amino Acid Metabolism after 15 min

In contrast to the rapid change in metabolite levels in the first 15 min, the following time period was relatively stable, with majority of metabolites staying within 2-fold difference between the consecutive time points (56 of 75 metabolites between 15 and 30 min, and 50 of 75 metabolites between 30 and 60 min, respectively).

The time period between 15 and 60 min after N-starvation was mainly characterized by the changes in amino acid composition. After passing the 15-min time point, all free amino acids sharply declined (Figure 3D). This result might be a direct consequence of the nitrogen source depletion, analogous to the above-described shutdown of purine synthesis. Surprisingly, though, the levels of some modified amino acids, such as methylated lysine and arginine, gradually increased (Figure 3E). Among these was also ergothioneine (Figure 3F), a sulphur-containing derivative of trimethyl histidine, widely believed to act as a physiological antioxidant [24]. Besides ergothioneine itself, we could also detect a sharp increase in hercynylcysteine sulfoxide, known to be a precursor of ergothioneine in *Neurospora crassa* [25]. Additionally, an S-methyl derivative of ergothioneine was first detected 60 min after N-starvation. No function has so far been reported for this compound.

Two tripeptides were detected in our data set—glutathione (only in its disulfide form, probably due to oxidation during the extraction procedure) and ophthalmic acid. In mouse liver, ophthalmic acid reportedly shares the biosynthesis pathway with glutathione, and exhibits elevated level under oxidative stress when glutathione is depleted [26]. In our experiment, however, the level of ophthalmic acid decreased sharply after N-starvation, while the level of glutathione remained stable (Figure 3G), suggesting a different regulation mechanism might take place in *S. pombe*.

## 3. Experimental Section

### 3.1. Strains and Culture Conditions

The fission yeast *S. pombe* heterothallic haploid 972 h^−^ wild-type strain [27] was used for the experiments. Cells were incubated in the EMM2 liquid medium [28] at 26 °C in a water bath with shaking. Cell concentration in the culture was measured by the CDA-500 particle counter (Sysmex, Kobe, Japan). For metabolome analysis, cells were grown to the log phase and at the concentration of 5 × 10^6^ cells/mL, 40 mL of culture was taken as 0 min sample. The rest of culture was N-starved by switching the medium with EMM2-N (EMM2 without NH_4_Cl) following the procedures previously described [7]. After N-starvation, cells were incubated again in the water bath at 26 °C, and after 15, 30, 60 min, 40 mL of culture was taken.

### 3.2. Microscopy

For cell length measurement, time course images were taken by using a DeltaVision Spectris restoration microscope (Applied Precision LLC, Issaquah, WA, USA) with a CH350L CCD camera (Photometrics, Tucson, AZ, USA). Cells were placed on a glass-bottomed culture dish (MatTek, Ashland, OR, USA) and covered with a slip of appropriate agar solid medium. For DAPI staining, cells were fixed with 2% glutaraldehyde for 10 min on ice, washed three times with phosphate buffered saline (PBS), and observed under a fluorescence microscope after staining with DAPI (25 µg/mL). Septation index was measured by counting septated cells in about 200 DAPI-stained cells.

### 3.3. Metabolome Sample Preparation

Metabolome samples were prepared by the procedure described previously [18]. Briefly, cells from cultures (40 mL/sample) were collected by vacuum filtration and immediately quenched in 25 mL of –40 °C methanol. Cells were harvested by centrifugation at −20 °C and a constant amount of internal standards (10 nmol of HEPES and PIPES) was added to each sample. Cells were disrupted using a Multi-Beads Shocker (Yasui Kikai, Osaka, Japan). Proteins were removed by filtering on an Amicon Ultra 10-kDa cut-off filter (Millipore, Billerica, MA, USA) and samples were concentrated by vacuum evaporation. Finally, each sample was resuspended in 40 µL of 50% acetonitrile and 1 µL was used for each LC-MS injection.

### 3.4. LC-MS Analysis

LC-MS data were acquired using a Paradigm MS4 HPLC system (Michrom Bioresources, Auburn, AL, USA) coupled to an LTQ Orbitrap mass spectrometer (Thermo Fisher Scientific, Waltham, MA, USA). LC separation was performed on a ZIC-pHILIC column (Merck SeQuant, Umeå, Sweden; 150 × 2.1 mm, 5 µm particle size). Acetonitrile (A) and 10 mM ammonium carbonate buffer, pH 9.3 (B) were used as the mobile phase, with gradient elution from 80% A to 20% A in 30 min and 100 µL/min flow rate. Mass spectrometer was operated in full scan mode with the scan range of 100–1,000 m/z. Each sample was analyzed twice, once in negative and once in positive ionization mode.

### 3.5. LC-MS Data Processing

Raw LC-MS data were analyzed by the MZmine 2 software [14]. The complete workflow and processing parameters are summarized in upplementary Table S2.

## 4. Conclusions

**Figure 4:**
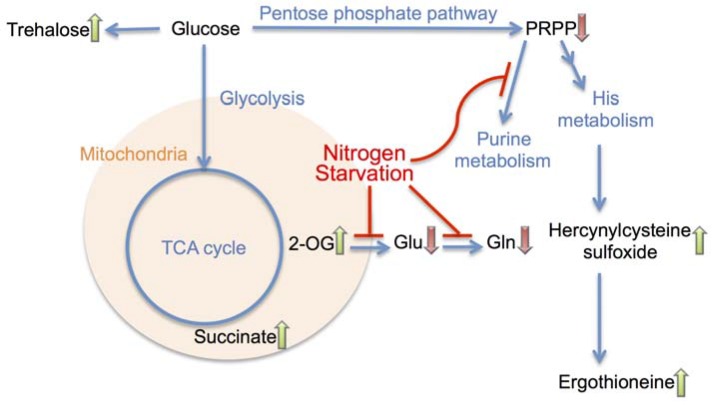
A summary diagram of metabolomic changes in *S. pombe* cells at the onset of nitrogen starvation.

In this study we characterized the metabolic response of *S. pombe* to N-starvation during the critical first hour, as schematized in Figure 4. Interestingly, a major remodeling of the cellular metabolome seemed to occur within the first 15 min, while later time points mostly exhibited continuation of the initial trends. The time interval of 15 min is quite short for gene expression and translation; we might thus suspect that a substantial part of the N-starvation response might be implemented by enzymes already present in the vegetative cell.

In the previously published transcriptomic and proteomic analyses, a significant overlap between stress response and N-starvation response was evident [5]. At the level of metabolome, this was reflected by the accumulation of trehalose and ergothioneine, both of which have protective functions. However, as we previously observed a similar accumulation of trehalose and ergothioneine under glucose starvation [19], such response seems to be rather non-specific. The rapid and steep accumulation of 2OG, on the other hand, seems to be specific to N-starvation and can be considered a promising biomarker of this condition. It is worth mentioning that the rapidly accumulated compounds (more than 5-fold in 15 min) such as 2OG, succinate and trehalose, do not contain nitrogen.

The depletion of nitrogen source led immediately to the reduction in purine biosynthesis intermediates. Among these intermediates was AICAR, a well-known activator for the cellular energy homeostasis regulator AMPK. AICAR is also a strong inhibitor of autophagy [29,30]. Since autophagy is known to be active after N-starvation [31], the decrease in AICAR level could act as a trigger for autophagy. The level of most free amino acids started decreasing only after 15 min from N-starvation, possibly due to the depletion of residual nitrogen source. On the contrary, the observed accumulation of several methylated amino acids brings up an interesting hypothesis that these metabolites might play some physiological role in the adaptation to N-starvation. The simplest explanation could be that these compounds represent a way to stock nitrogen at the metabolic level.

Our key results (increase in 2OG and trehalose, decrease in amino acids) are consistent with the nitrogen starvation metabolome results in *S. cerevisiae* [32]. On the other hand, the quick depletion of purine intermediates such as AICAR was not previously described. Also, ergothioneine accumulation seems to be specific to *S. pombe*, as budding yeast does not produce this compound.

Taken from a wider perspective, the metabolites described in this study shed some light on the immediate—and otherwise unnoticeable—intracellular response to N-starvation. In the future, monitoring the levels of these compounds can provide new insights when diagnosing *S. pombe* strains that show defects in entry to, maintenance of, or exit from N-starvation.

## Supplementary Material

Supplementary materials can be accessed at: http://www.mdpi.com/2218-1989/3/4/1118/s1.

The raw LC-MS data in mzML format was submitted to the MetaboLights repository (URL: http://www.ebi.ac.uk/metabolights) under accession number MTBLS67.

Supplementary File 1 Supplementary (ZIP, 564 KB)
