# Supplementary material for: Metabolomic Analysis of Fission Yeast at the Onset of Nitrogen Starvation
Source: Metabolites. 2013 Dec 13;3(4):1118–29. doi: 10.3390/metabo3041118 (PMC3937841; doi:10.3390/metabo3041118)

Supplementary Materials

Figure S1. MS/MS analysis of peaks identified as FGAR (A), SAICAR (B) and hercynylcysteine sulfoxide (C).

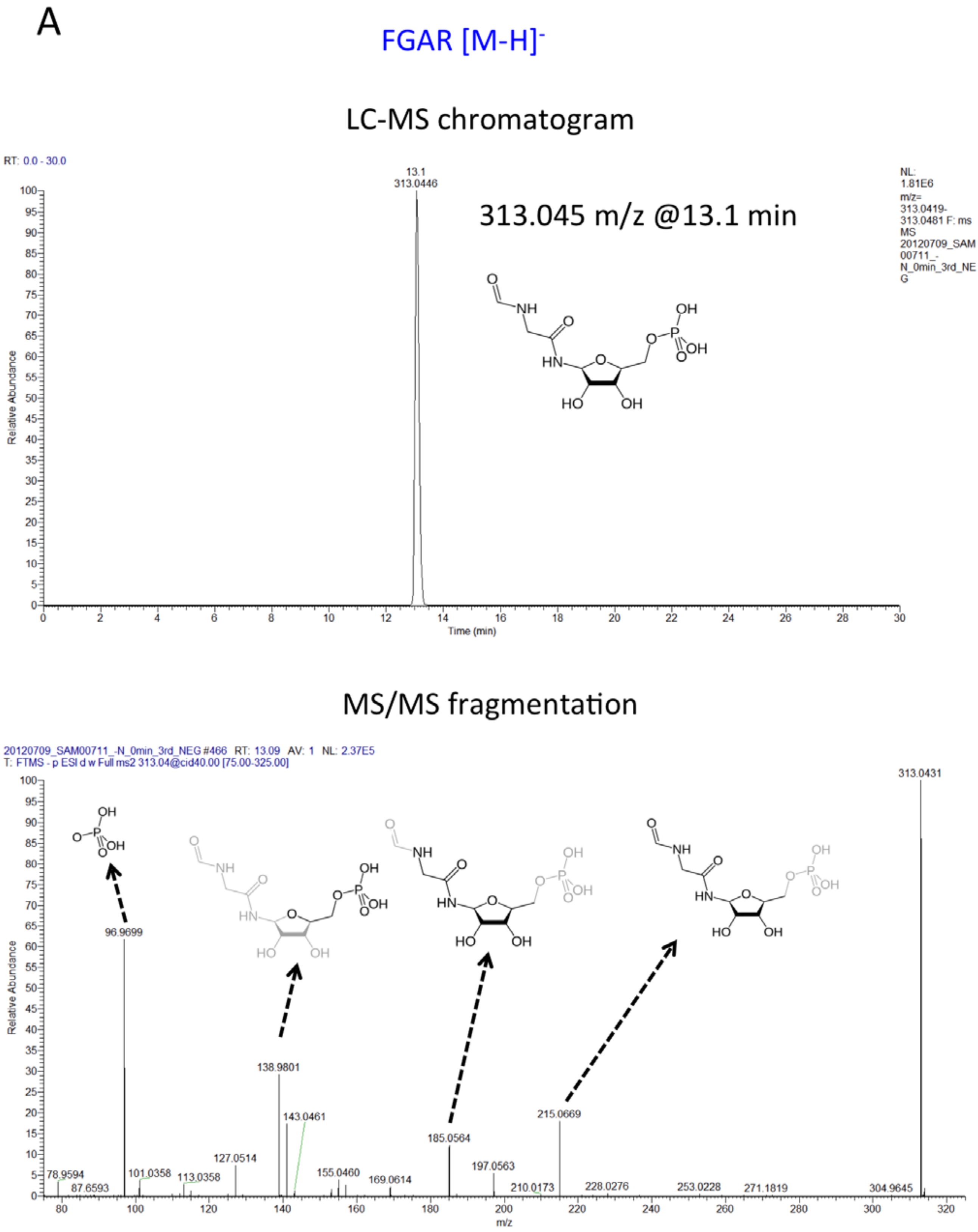

B

SAICAR [M-H]$^-$

LC-MS chromatogram

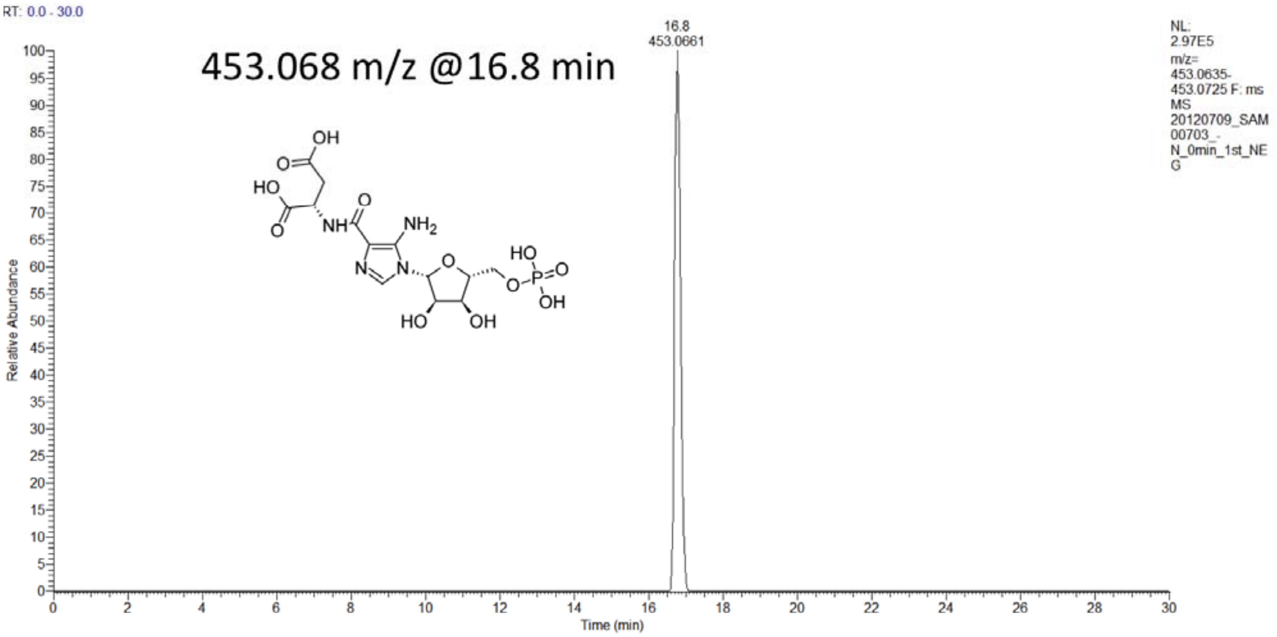

MS/MS fragmentation

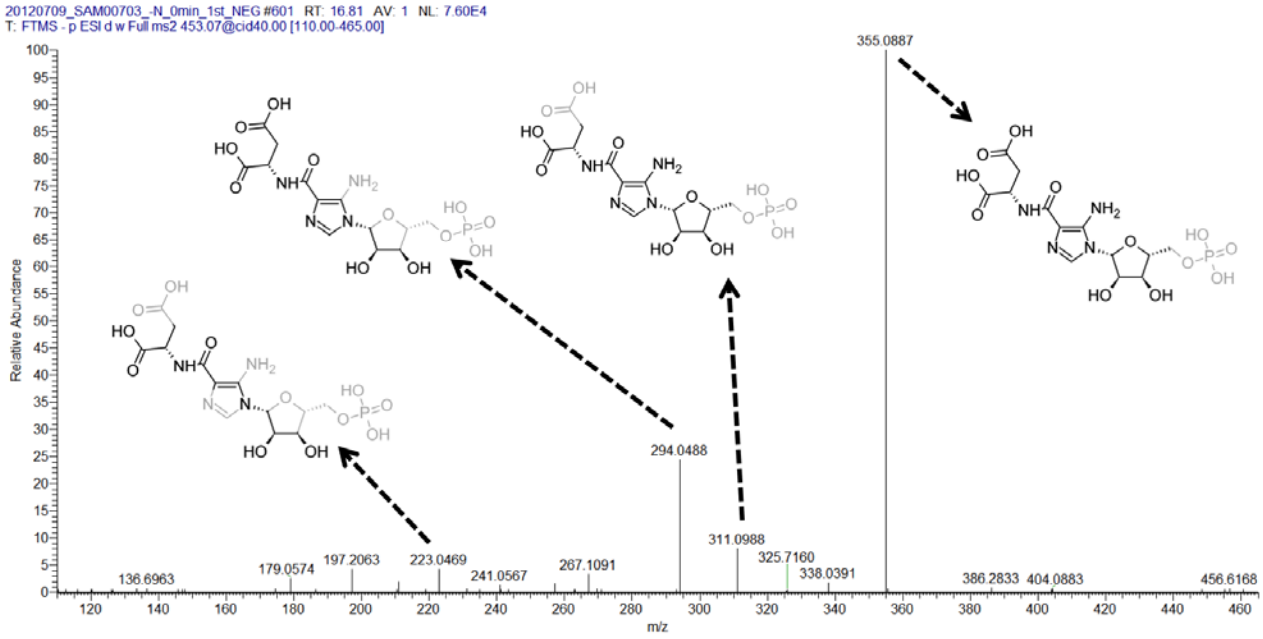

C

Hercynylcysteine sulfoxide $[M+H]^+$

LC-MS chromatogram

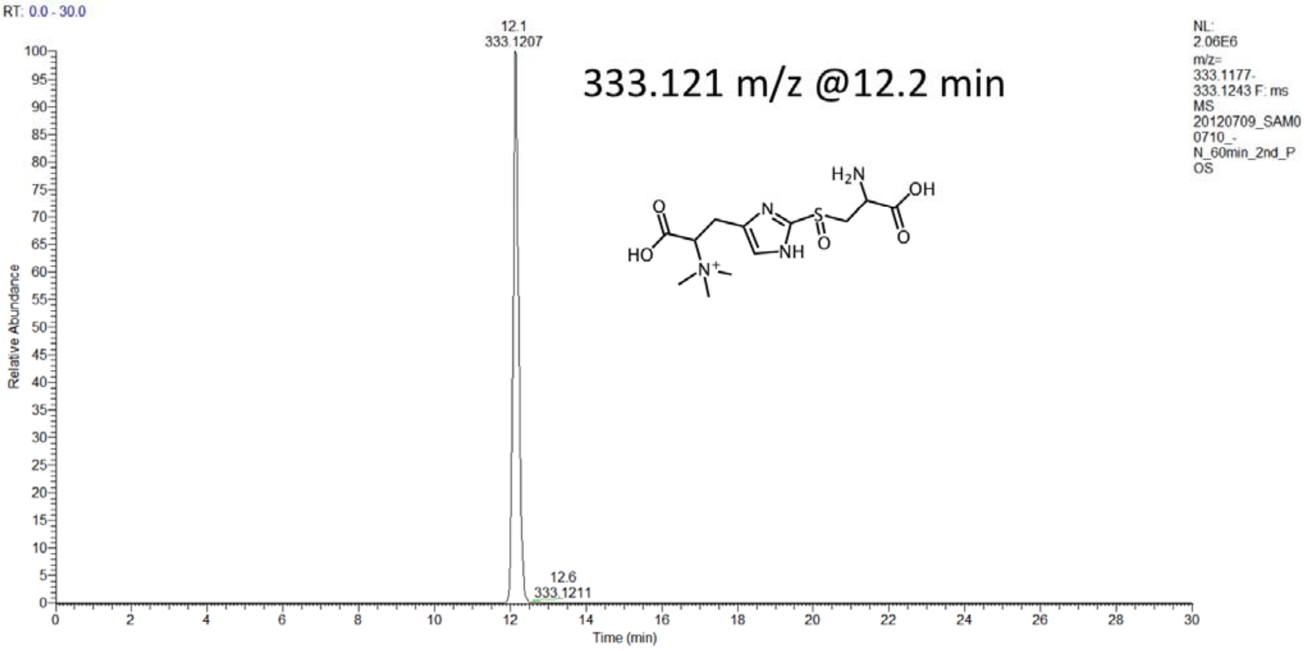

MS/MS fragmentation

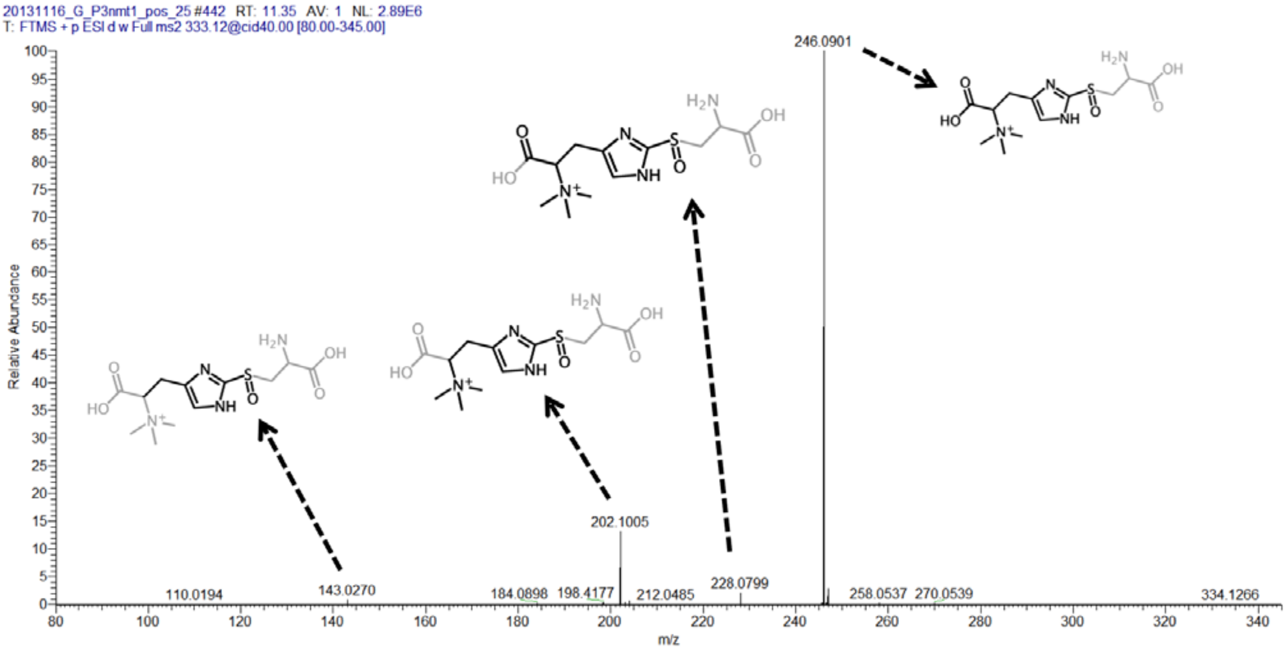

Supplement: Supplementary File 1 — Supplementary (ZIP, 564 KB) [file metabolites-03-01118-s001.zip › metabolites-03-01118-supplementary/Figure S1.pdf]
